# A bird-inspired artificial intelligence framework for advanced large text summarization

**DOI:** 10.3389/frai.2026.1703769

**Published:** 2026-03-17

**Authors:** Binxu Huang, Anasse Bari

**Affiliations:** Department of Computer Science, Courant Institute of Mathematical Sciences, New York University, New York, NY, United States

**Keywords:** artificial intelligence, bird flocking, extractive summarization, factual consistency, hybrid ranking, knowledge graphs, LLM hallucination, natural language processing

## Abstract

We introduce a biologically inspired bird-flocking experimental framework for text summarization that identifies the most salient sentences using contextual information, sentence position, and thematic relevance. The bird-flocking-inspired algorithm, combined with large language models (LLMs), generates summaries with greater factual accuracy. The algorithm ensures source faithfulness by preventing the generation of new, unsupported information, thereby mitigating the risk of model hallucination by grounding the summary exclusively in the original text. While large language models (LLMs) achieve remarkable fluency in abstractive summarization, they frequently hallucinate generating plausible but unsupported content. We introduce a bio-inspired bird-flocking framework that addresses this limitation by serving as a preprocessing step for LLM-based summarization. Our method identifies the most salient, source-faithful sentences using contextual information, sentence position, and thematic relevance, providing LLMs with factually grounded input that constrains generation to verified content. Experimental results show that our methodology consistently produces concise and factually correct summaries, as experimented with the commonly used quality measurement scores. The framework provides a mechanism for text summarization that incorporates unified stop-word control, collocation recognition with synonym expansion, attention combination with fallback, score normalization between global and local saliency, and an unsupervised learning bio-inspired Flock-by-Leader text clustering algorithm. These components contribute not only to improved consistency and diversity of the summary, but also to reduced hallucinations in text summarization. The algorithms and experimental framework proposed in this study serve as an efficient preprocessing step that complements both conventional and generative AI-based text summarization methods. The framework produces a well-structured intermediate representation of the source document, which is then provided to the LLM to generate the final summary. Across over 9,000 long-form documents in healthcare and energy, our framework consistently outperforms a major large language model baseline, with gains of 7.28% in ROUGE-1, 6.19% in ROUGE-L, and 45.28% in entity coverage.

## 1 Introduction

In today's information age, the world is drowning in text. The volume of unstructured content across the internet, scientific literature, legal documents, and corporate communications has grown exponentially, making it impractical to manually manage and summarize large-scale documents. Automatic text summarization (ATS) emerges as a critical subfield of natural language processing (NLP) and artificial intelligence (AI), computationally condensing extensive text documents into shorter, coherent representations of key information (Allahyari et al., 2017; Gambhir and Gupta, 2017). The fundamental challenge of information overload and the inability of human beings to process it all, often termed “Future Shock” (Toffler, 1970), has required more use of automatic text summarization algorithms, especially for large text.

While modern abstractive models such as GPT-4 achieve remarkable fluency, they frequently suffer from hallucination which generates plausible text unsupported by the source (Ji et al. 2023) and OpenAI (2025). This work addresses this limitation not by replacing LLMs, but by augmenting them. We present BMAPS, a Bird Multi-modal Automated Processing and Summarization framework that identifies salient, source-faithful sentences to provide LLMs with factually grounded input, and can help mitigating hallucination risk. Its purpose is to provide generative models with factually grounded, salient sentences extracted directly from the source text as pre-processing step for summary extraction by LLMs.

This paper makes four key contributions to the field of text summarization. First, we propose BMAPS, a bio-inspired framework that aims at mitigating hallucination in LLM-based summarization by grounding generation in factually salient source sentences. Second, we introduce a hybrid ranking strategy that combines global, local, and topic saliency, attentive multimodal feature fusion, and fallback mechanisms. Third, we adapt the Flock-by-Leader data clustering algorithm originally designed for general document clustering to enforce topical diversity in summaries without requiring a predefined number of clusters. Finally, we validate our approach through large-scale experiments on over 9,000 documents that demonstrated consistent improvements of 7.28% in ROUGE-1, 6.19% in ROUGE-L, and 45.28% in entity coverage over strong LLM baselines.

## 2 Related works

Automatic Text Summarization research traces its roots to the seminal work of (Luhn 1958), who proposed extracting sentences based on the high frequency of words in them. (Edmundson 1969), in turn, later added the cue phrases, title words, and sentence location as clues to the feature set. Another major step in extractive summarization was achieved with graph-based ranking algorithms, such as TextRank (Mihalcea and Tarau, 2004), which represent sentences as graphs and use centrality metrics to identify the most salient ones. This method is a good way to understand that significant sentences are those highlighted by other important sentences. Abstractive summarization came into the limelight with the development of deep learning. Early neural models employed recurrent neural networks (RNNs) with attention mechanisms to generate summaries word-by-word (Ramesh Nallapati, 2016; Rush et al., 2015). One of the main innovations was the pointer-generator network, which combined abstractive generation with the ability to point to and copy words from the source text, alleviating problems with out-of-vocabulary words and enhancing the factual accuracy of the model (See et al., 2017).

The current state of the art is dominated by large, pre-trained transformer models such as OpenAI's GPT series (Brown et al., 2020), DeepSeek (DeepSeek-AI, 2024), PEGASUS (Zhang et al., 2020), and Meta's BART (Lewis et al., 2020) and Text Summarization with Pretrained Encoders (Liu and Lapata, 2019). These models are fine-tuned for summarization and achieve remarkable fluency and contextual understanding. In more recent work, some research has experimented with bio-inspired and swarm intelligence algorithms (Nayyar et al., 2018) and has applied ideas from nature to optimize sentence selection (Rautray and Balabantaray, 2017). Nevertheless, even though large abstractive models have been successful, they still encounter major challenges. These models often suffer from factual inconsistency, a phenomenon commonly known as “hallucination” (Ji et al., 2023), where a model generates plausible-sounding text that coud be factually incorrect or unfaithful to the source data (OpenAI, 2025). These limitations, together with positional bias in lengthy documents (Liu et al., 2024) and high computational cost, remain significant challenges. We are right on the boundary of these trends, offering a hybrid, bio-inspired approach that aims to reap the benefits of both extractive and abstractive paradigms while aiming to provide enhancements to help make summaries more factual and correct.

## 3 Methods

We propose a bio-inspired AI framework for text summarization, the Bird Multi-modal Automated Processing and Summarization (BMAPS) AI framework, an end-to-end computational pipeline that compresses unstructured scholarly texts in any format into a structured knowledge representation. The system architecture combines lexical, semantic, topical, and visual information to detect the most salient content and synthesize it. Here, we discuss the modules of this framework and highlight its improvements.

### 3.1 Module one: document parsing and canonicalisation

**Figure 1:**
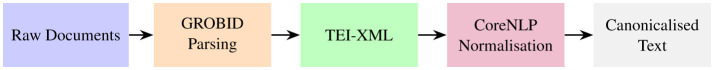
Workflow for document parsing and cannibalization.

To encode the raw documents into a machine-readable format, we used the GROBID (0.8.2) machine learning library (Lopez, 2009) to extract the logical structure of the documents, producing TEI-XML files that describe the document's elements, including title, abstract, and sectioned body text. The text mined in each section was then subjected to a normalization pipeline with Stanford CoreNLP(4.5.10) (Manning et al., 2014), which involved tokenisation, part-of-speech tagging, and lemmatisation. To cope with terminological heterogeneity, the set of canonical terms and their variations was represented as a domain-specific knowledge base, and an automaton for search was built using the Aho-Corasick algorithm (Aho and Corasick, 1975). This made it possible to perform a single-pass standardization of all known multi-word entities within the text, ensuring terminological consistency in future analyses. Figure 1 provides a visual overview of this workflow.

### 3.2 Module two: advanced text preprocessing

Following canonicalisation, we use a superior text preprocessing module. This module builds on a typical NLP pipeline in several ways. First, it is a centralized stop-word filter that uses a specialized StopWordManager to ensure document consistency. Second, it carries out fine-grained part-of-speech filtering, keeping nouns, verbs, and adjectives and deleting less informative tokens. Lemmas are made lowercase, and punctuation is removed. Third, the preprocessor detects common collocations by searching for bigrams and trigrams that appear with a user-defined frequency; the phrases are concatenated with underscores to maintain multi-word phrases (e.g., “lung cancer” is transformed into “lung_cancer”). Fourth, an Aho-Corasick automaton is used to efficiently match multiple patterns against a domain dictionary to standardize domain-specific entities. Lastly, optional data augmentation substitutes certain tokens with synonyms selected from WordNet—a word lexicon database that stores words according to semantic categories and synonyms in so-called synsets—to further expand the vocabulary base (Fellbaum, 1998). The combination of methods results in a clean stream of contextually rich tokens, which is visible to the downstream feature extraction.

### 3.3 Module three: multi-modal feature engineering

By engineering features across four modalities, we built an overall representation of the document's content. Lexical properties were obtained by calculating the term frequency divided by document frequency (*TF*−*IDF*) vectors. We introduced sublinear *TF* scaling to avoid the dominance of common words, where the value for a term *t* in a sentence *s* is given by


$$\begin{array}{l} \text{TF-IDF}\left(t,s\right)=\left(1+\log\left(\text{t}{\text{f}}_{t,s}\right)\right)\times \log\left(1+\frac{N}{n_{t}}\right) \end{array}$$ (1)

In which tf_*t, s*_ is the occurrence frequency of term *t* in sentence *s*, *N* is the total number of sentences, and *n*_*t*_ is the number of sentences that include term *t*. Semantic features were generated using the sentence transformer model known as all-MiniLM-L6-v2, developed by (Reimers and Gurevych 2019), mapping each sentence to a 384-dimensional embedding (**v**_sem_). The Latent Dirichlet Allocation (LDA) was used to model topical features (Blei et al., 2003) and returned a document-level topic distribution vector (**v**_topic_). Regarding visual features, the Contrastive Language-Image Pre-Training (CLIP) model was used to embed images and tables into the same semantic space as visual features (Radford et al., 2021).

### 3.4 Module four: attentive multi-modal feature fusion

This module integrates the diverse sentence representations included in the lexical (TF-IDF), semantic (S-BERT), topical (LDA), and visual features (CLIP) language into a unified vector space for downstream ranking. A naive concatenation or averaging of these features is suboptimal because different modalities contribute unevenly to a sentence's saliency depending on the context. To address this, we employ an Attentive Feature Fusion mechanism that dynamically assigns importance weights to each modality.

Since the raw feature vectors originate from different embedding spaces with varying dimensions, we first project each modality *m* ∈ {tfidf, sem, topic, visual} into a shared latent space of dimension *d* = 512. This is achieved via modality specific linear projection layers:


$$\begin{array}{l} {\text{p}}_{m}={\text{W}}_{m}{\text{v}}_{m}+{\text{b}}_{m} \end{array}$$ (2)

In this equation:

- ${\text{v}}_{m}\in {\mathbb{R}}^{d_{in}}$ denotes the raw input feature vector for modality *m* (e.g., the 384-dimensional semantic embedding generated in Module 3).
- ${\text{W}}_{m}\in {\mathbb{R}}^{512\times d_{in}}$ is the learnable projection matrix that maps the input vector from its original dimensionality (*d*_*in*_) to the target unified dimension (*d* = 512).
- **b**_*m*_ is the corresponding bias term.

The core of this module is the computation of the attention scores **α**. We utilize a multi-layer perceptron (MLP) as a scoring function. The projected vectors are concatenated to form a comprehensive context vector, which is then fed into the MLP. The MLP learns to evaluate the relative confidence of each modality based on the input context:


$$\begin{array}{l} \alpha =\text{softmax}\left(\text{ML}{\text{P}}_{\text{attention}}\left(\left[{\text{p}}_{\text{tfidf}};{\text{p}}_{\text{sem}};{\text{p}}_{\text{topic}};{\text{p}}_{\text{visual}}\right]\right)\right) \end{array}$$ (3)

Here, **α** represents a vector of weights, such that ∑α_*m*_ = 1. The final attended vector (**v**_attended_) is computed as the interaction-weighted sum of the projected features:


$$\begin{array}{l} {\text{v}}_{\text{attended}}=\underset{m}{\sum }\alpha_{m}\cdot {\text{p}}_{m},\;{\text{v}}_{\text{ultimate}}=\text{ML}{\text{P}}_{\text{final}}\left({\text{v}}_{\text{attended}}\right) \end{array}$$ (4)

This fused representation (**v**_ultimate_) encapsulates the holistic saliency of the sentence.

To ensure system stability, we incorporate a Robustness Protocol. If a modality is missing (yielding a null **v**_*m*_), the attention mechanism masks it by assigning a zero weight. In extreme cases of numerical instability, a Semantic Fallback Mechanism bypasses fusion and defaults to the semantic embedding (**v**_sem_) to ensure downstream ranking always receives a valid vector.

### 3.5 Module five: knowledge graph construction

To go beyond summarization toward structured knowledge extraction, we use Stanford CoreNLP to identify named entities (NER) and apply dependency parsing to extract relationships between them (Manning et al., 2014). The resulting tuples are compiled into (subject, relation, and object) triples that are later used for salience ranking.

## 4 Hybrid ranking and clustering algorithm

### 4.1 Stage 1: hybrid graph-based ranking

The essence of our ranking model is the principle of integrated saliency, which considers the importance of sentences from multiple perspectives. For a given set of sentences {*s*_1_, …, *s*_*n*_}, we first construct a fully connected, weighted similarity graph *G* = (*V, E*), where *V* is the set of sentences. The weight of an edge (*s*_*i*_, *s*_*j*_) is the cosine similarity between their ultimate vectors:


$$\begin{array}{l} w_{ij}=\cos\left({\text{v}}_{i},{\text{v}}_{j}\right)=\frac{{\text{v}}_{i}\cdot {\text{v}}_{j}}{\|{\text{v}}_{i}∥∥{\text{v}}_{j}∥}. \end{array}$$ (5)

On this graph we compute scores based on the TextRank algorithm, an iterative voting mechanism (Mihalcea and Tarau, 2004). The score for a sentence *s*_*i*_ is updated based on the scores of sentences that link to it until convergence:


$$\begin{array}{l} \text{TR}\left(s_{i}\right)=\left(1-d\right)+d\underset{s_{j}\in \text{In}\left(s_{i}\right)}{\sum }\frac{w_{ji}}{\underset{s_{k}\in \text{Out}\left(s_{j}\right)}{\sum }w_{jk}}\text{TR}\left(s_{j}\right), \end{array}$$ (6)

The value of *d* is the damping factor (set to 0.85) and In(*s*_*i*_) is the set of inbound neighbors of *s*_*i*_. We calculate three different scores, namely, global centrality, local centrality, and thematic relevance, and sum all these scores to create the final saliency score. Min-max scaling of each component to the unit interval is performed before aggregation to ensure that no single measure supersedes the aggregate. We apply weights of 0.4, 0.3, and 0.3 to the normalized global, local and thematic scores, respectively, and introduce a section-type weight *W*_sec_(*s*_*i*_) that assigns higher importance to sentences appearing in critical sections (Abstract, Introduction, Results, and Conclusion) than those in generic body paragraphs. The final hybrid score, with weights determined through grid search (see Section 5.4), is computed as $S_{\text{final}}\left(s_{i}\right)=W_{\text{sec}}\left(s_{i}\right)\left[0.4S_{\text{global}}^{\prime }\left(s_{i}\right)+0.3S_{\text{local}}^{\prime }\left(s_{i}\right)+0.3S_{\text{thematic}}^{\prime }\left(s_{i}\right)\right]$.

### 4.2 Stage 2: diversity enhancement via Flock-by-Leader algorithm

We apply the Flock-by-Leader approach that is based on the flocking of birds-agents in a high-dimensional virtual space to cluster sentences without prior knowledge about how many clusters there will be (Bellaachia and Bari, 2012; Cui et al., 2006).

**Figure 2:**
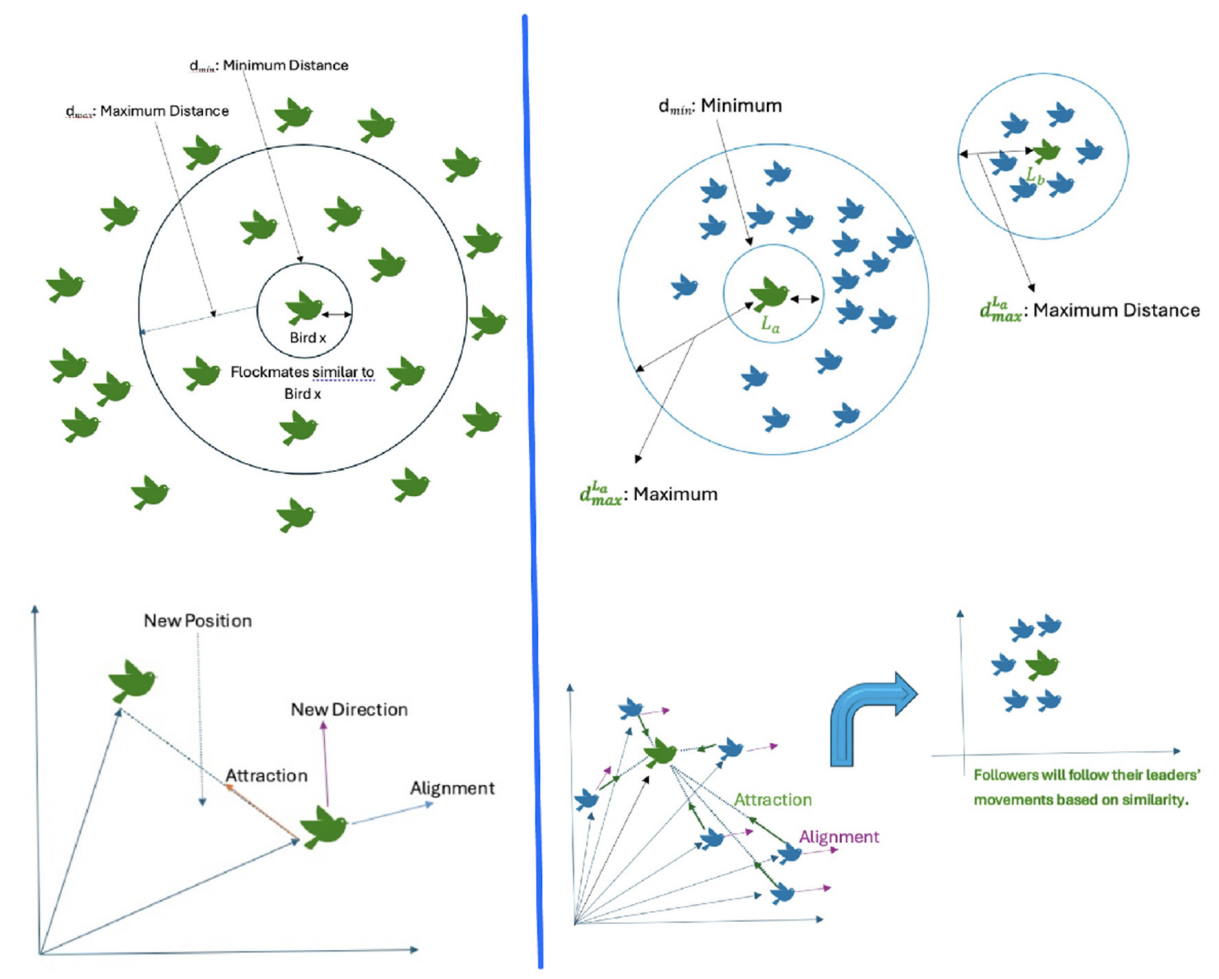
A visual representation of the flocking algorithm. The **left** panel shows the basic model with attraction and alignment forces. The **right** panel illustrates the Flock-by-Leader strategy, where agents form distinct clusters around leaders (Bellaachia and Bari, 2012).

In our enhanced implementation, the number of nearest neighbors *k* is set to $\max\left(k_{\min},\left\lceil \sqrt{n}\right\rceil \right)$ for *n* sentences, and clustering of ranked sentences proceeds in three phases: initial flocking, iterative refinement and cluster merging. Each bird-agent is repeatedly exhibited in the cluster with the nearest centroid during refinement until assignments stabilize. In a final pass, adjacent clusters with centroid larger than an experimental similarity threshold, τ, are combined. Adjacent clusters whose centroids exceed a similarity threshold τ are merged in a final pass. The final summary is constructed by selecting sentence representatives from each cluster and ordering them according to their original position. This self-organized clustering algorithm is inspired by the collective behavior of natural swarms of agents (Reynolds, 1987). We adopt the Flock-by-Leader approach (Bellaachia and Bari, 2012); see Algorithm 2; see Figure 2.

Algorithm 1 **Hybrid sentence ranking.**

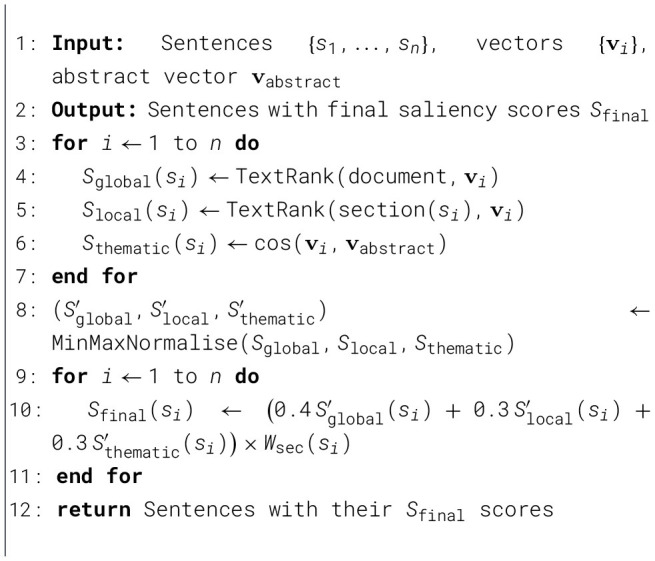

Algorithm 2 **Diversity-enhanced summary generation with Flock-by-Leader [adapted from (Bellaachia and Bari 2012)].**

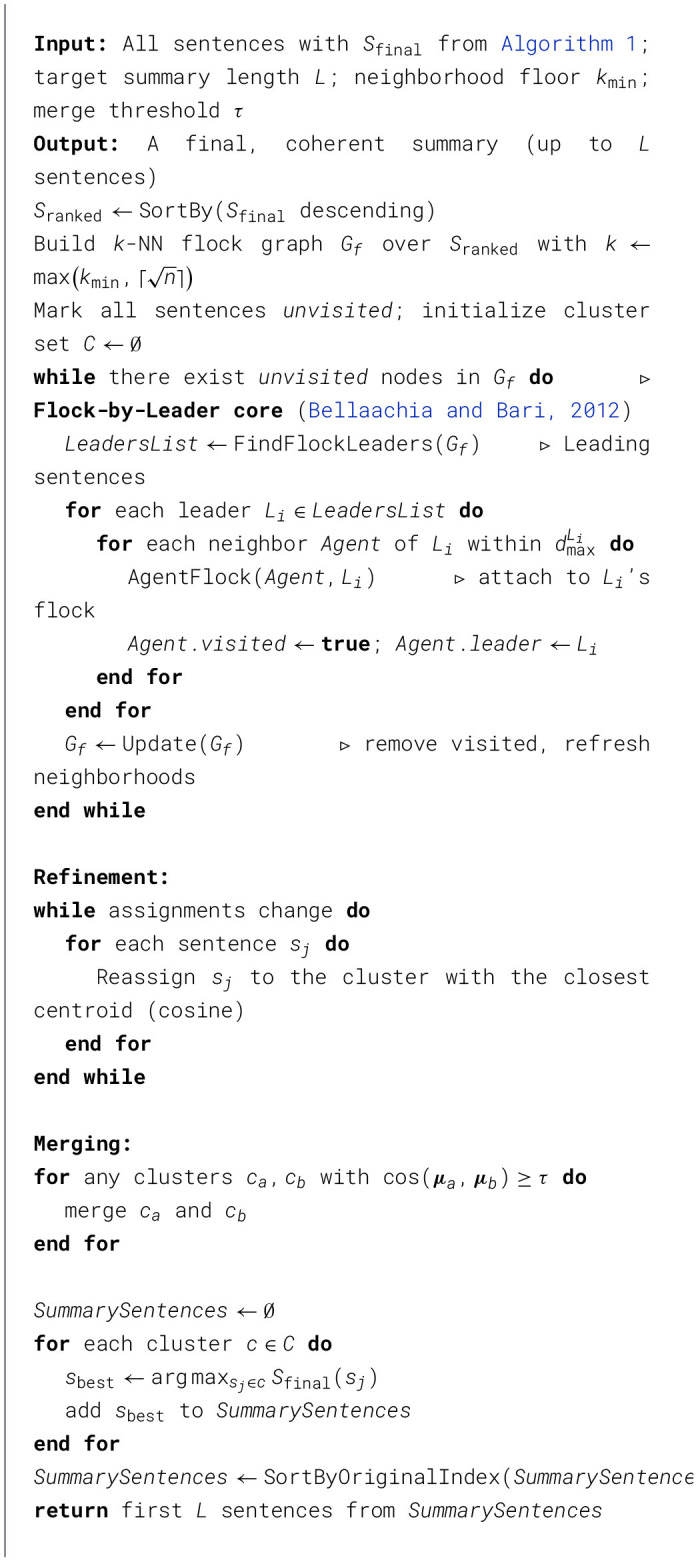

## 5 Experiments

We conducted two rounds of experiments: an ablation study to determine the role of key algorithmic elements and a head-to-head competition against a large language model (LLM) baseline ChatGPT-4 api. The preprocessing techniques and the algorithms mentioned in the previous section were all part of the experiments.

### 5.1 Dataset and experimental setup

The evaluation corpus contains 9,069 publicly available, long-form English documents collected from open-access scientific repositories and technical report sources across the healthcare and energy domains. In particular, 2,210 documents were drawn from the nuclear energy subfield of Low Energy Nuclear Reactions (LENR), sourced from our lab's public LENR Dashboard repository (https://lenrdashboard.com) (LENR Dashboard, 2026). The remaining 6,859 documents were collected from healthcare literature, focusing on multiple cancer types and including open-access publications from sources such as The Oncologist (Oxford Academic, 2025) and the U.S. National Library of Medicine (2025). Overall, the dataset includes peer-reviewed academic papers and technical reports authored by clinicians, researchers, and LENR experts. All documents were processed using the same preprocessing pipeline and feature-extraction components described in Section 3 to ensure a fair and consistent comparison across experimental settings. For comparison, we evaluated our method against a large language model (LLM) summarization baseline, namely ChatGPT 4. The model was prompted to generate summaries directly from the full document text, with output length normalized to match that of our experimental framework.

### 5.2 Evaluation metrics

In order to measure the quality of the summaries produced, we used the most common Recall-Oriented Understudy for Gisting Evaluation (ROUGE) metrics (Lin, 2004). ROUGE operates through the comparison of a machine-generated summary to a single or multiple human-written reference text. The F1-scores of the three most used metrics are reported by us:

- ROUGE-1 measures the overlap of unigrams (individual words).
- ROUGE-2 measures the overlap of bigrams (pairs of adjacent words), which reflects phrase-level similarity.
- ROUGE-L measures the longest common subsequence, which captures sentence-level structural similarity.
- Diversitys assesses the semantic variety within the generated summary to penalize redundancy. We calculate it as the average pairwise dissimilarity (specifically, 1 − cosine similarity) between the sentence embeddings of all sentences in the summary. Higher values indicate that the summary covers a broader range of topics from the source document.

Higher ROUGE scores generally indicate a better-quality summary in terms of content overlap with the reference text.

### 5.3 Clustering configurations

We tested the three design options: (i) introducing a step of *k*-means clustering before the flocking algorithm, (ii) using sentences of the smallest related clusters (inverted *k*-means), and (iii) changing the number of leaders during the flocking algorithm. Table 1 summarizes the averaged results across all documents; full results for all tested *k* values are provided in a Supplementary Table.

**Table 1:** Ablation study results showing the effect of *k*-means clustering, inverted *k*-means and varying flocking leaders.

**Configuration**	**ROUGE-1**	**ROUGE-2**	**ROUGE-L**	**Diversity**
Baseline (no clustering)	0.412	0.198	0.355	0.78
*k*-means + flocking (best *k* = 3)	0.385	0.171	0.321	0.62
Inverted *k*-means (best *k* = 3)	0.320	0.115	0.250	0.85
Weighted + flocking (2 leaders)	0.435	0.215	0.388	0.81
**Weighted + flocking (3 leaders)**	**0.451**	**0.228**	**0.405**	**0.83**
Weighted + flocking (4 leaders)	0.446	0.221	0.397	0.84

Best-performing configuration in bold.

The application of the *k*-means step consistently led to lower ROUGE scores than the baseline, presumably because feature diversity was lost before flocking. Inverted k-means enhanced diversity at the expense of seriously damaging accuracy. The weighted ranking + flocking setup, particularly the leader weighted solution offered the most desirable compromise between accuracy and diversity by yielding the highest ROUGE scores and competitive diversity.

### 5.4 Weight selection

The weights for global, local, and thematic scores (0.4, 0.3, and 0.3) were determined through grid search on a held-out development set of 500 documents. We evaluated all combinations of weights in {0.2, 0.3, 0.4, 0.5} subject to the constraint that weights sum to 1.0. Table 2 shows selected configurations.

**Table 2:** Grid search results for saliency weight selection.

**Global**	**Local**	**Thematic**	**ROUGE-1**	**ROUGE-L**
0.33	0.33	0.33	0.438	0.391
0.5	0.25	0.25	0.442	0.396
**0.4**	**0.3**	**0.3**	**0.451**	**0.405**
0.3	0.4	0.3	0.445	0.398
0.3	0.3	0.4	0.440	0.393

The selected configuration is shown in bold.

The configuration (0.4, 0.3, and 0.3) achieved the highest ROUGE scores, suggesting that global centrality is slightly more informative than section-level or thematic signals, while all three contribute meaningfully to final sentence selection.

### 5.5 Comparison with LLM baseline

Our most successful configuration (weighted + bird flocking algorithm with 3 leaders) as the preprocessing step to the LLM was compared to the LLM summarization baseline (ChatGPT-4 API). As a fair comparison, the target length of the summary of each system was adjusted on the length of the source document. This will help avoid bias in the results caused by output verbosity and make the evaluation depend on the quality of the content. The rating was based on 9,069 documents across healthcare and energy as mentioned in the previous sections.

Overall, our framework, used as a preprocessing step to the LLM, yields measurable improvements while reducing hallucinations by restricting summaries to sentences from the original text. The results are summarized in Table 3.

**Table 3:** Overall performance between our model and the LLM baseline on 9,069 documents, with summary lengths normalized for fair comparison.

**Metric**	**Preprocessing + LLM (Mean)**	**LLM baseline (Mean)**	**Relative improvement (%)**
ROUGE-1 F1	0.0516	0.0481	+7.29
ROUGE-L F1	0.0326	0.0307	+6.19
Entity coverage	0.0077	0.0053	+45.28
Flesch reading ease	20.0380	20.8770	−4.02

Relative improvement is computed as a percentage change from the baseline.

#### 5.5.1 End-to-end pipeline overview

**Figure 3:**
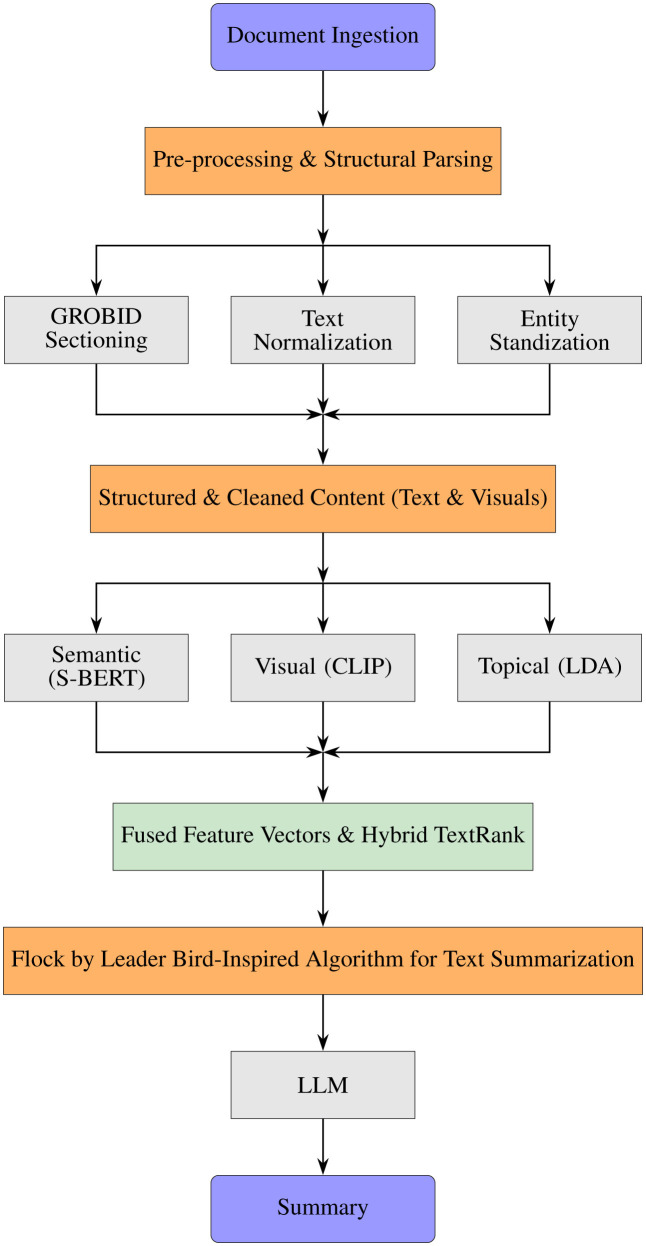
The end-to-end BMAPS pipeline, illustrating the workflow from raw PDF document ingestion to the generation of a structured summary.

The five modules described above form an integrated workflow that transforms raw documents into a structured representation suitable for ranking, clustering, and summarization. In particular, the pipeline begins with ingestion and structural parsing, proceeds through normalization and entity standardization, and then constructs multi-modal sentence features that support hybrid saliency scoring and diversity-aware selection. Figure 3 summarizes this end-to-end process and highlights how feature engineering and fusion feed into the downstream ranking and Flock-by-Leader stages prior to LLM-based summary generation.

## 6 Discussion

Our experimental findings show that the proposed model, which uses a bio-inspired flocking algorithm for weighted ranking, is much more effective than the LLM baseline. The best setup which consisted of three leaders had the highest scores on all ROUGE measures in generating relevant and correct summaries due to establishing the salient sentences with the help of a multi-faceted scoring system and second, providing topical diversity with the help of bio-inspired data clustering. The score normalization and section-type weighting eliminates the dominance of one specific component, making the selections more equal. The Flock-by-Leader algorithm adapts the neighborhood size based on the sentences in the document in question, then refines and merges to generate coherent clusters. This procedure draws inspiration from natural flocking behavior, where agents follow simple local rules and self-organize into cohesive groups (Reynolds, 1987). The viability of this paradigm is reinforced by the successful application of similar bio-inspired models in other complex domains (Bellaachia and Bari, 2012).

The primary advantage of our approach is that it complements state-of-the-art LLMs rather than competing with them. LLMs excel at generating fluent, abstractive summaries, but they are prone to hallucinations (Ji et al., 2023). Our framework addresses this by serving as a preprocessing filter that identifies the most factually salient sentences before LLM processing. This constrains generative models to work with verified, source-grounded content. In this way, we leverage the strengths of both approaches: our bio-inspired extraction ensures factual fidelity, while downstream LLMs provide fluent synthesis of the pre-selected material.

Our model has a relatively advanced ranking mechanism compared to traditional ranking methods (Mihalcea and Tarau, 2004). Rather than using a single centrality measure, it combines global centrality, local (section-specific) significance as well as thematic relevance to the abstract. Additionally, the Flock-by-Leader clustering phase focuses specifically on redundancy, resulting in a more thorough and balanced coverage of the document's most important points. The various engineering changes that we have made, such as consolidated stop-word handling, enhanced preprocessing with collocation and synonym expansion, attention-fusion fallback, and dynamic cluster refinement, all contribute to the effectiveness of the experimental framework presented in this study.

Our framework has limitations. First, while we conducted a preliminary human evaluation with a small number of members from our lab, future work should adopt a larger and more structured evaluation protocol, including assessments from domain experts, to more rigorously measure factuality, coherence, and practical usefulness. In addition, several components rely on upstream tools and pretrained models (e.g., embedding models, topic modeling, NER, and dependency parsing), meaning that performance may vary depending on domain-specific terminology and the quality of these external modules. The flocking algorithm also includes hyper-parameters such as the minimum neighborhood size, merge threshold, and number of refinement iterations, which may require tuning across different document types for optimal performance. Second, although synonym expansion can improve lexical coverage, it may introduce subtle shifts in meaning if the replacement probability is not carefully controlled. Finally, since our experiments were limited to English text, future work will extend the framework to additional languages and domains. We plan to address these limitations in the next version of our experimental framework by expanding expert-driven evaluation, improving domain adaptation, and introducing more systematic hyperparameter tuning.

### 6.1 Computational considerations and scalability

Given the multi-stage pipeline and large evaluation corpus (over 9,000 long-form documents), computational efficiency and scalability are key considerations. Runtime is mainly driven by (i) sentence-level representation computation for semantic similarity and clustering and (ii) neighborhood-graph operations in the flock-by-leader stage. Most steps scale approximately linearly with the number of sentences, while flocking remains tractable due to a sparse graph with bounded neighborhood size.

The system is naturally parallelizable at the document level. Using two NVIDIA H100 GPUs, processing the full corpus required approximately 1 h, whereas the external LLM baseline (e.g., GPT-4 API) required about 4 h due to latency and rate limits. These results indicate that the pipeline is suitable for large-scale summarization and can be further accelerated via batching, approximate nearest-neighbor search, and GPU-based embedding inference.

## 7 Conclusion

Our work presents a text summarization framework inspired by bird flocking behavior. Our experimental results suggest that this approach serves as an effective preprocessing step for LLM-based summarization, producing more informative summaries and potentially reducing hallucinations in generated outputs.

## Data Availability

The original contributions presented in the study are included in the article/supplementary material, further inquiries can be directed to the corresponding author.
